# *Anisakis* Allergy: Is Aquacultured Fish a Safe and Alternative Food to Wild-Capture Fisheries for *Anisakis simplex*-Sensitized Patients?

**DOI:** 10.3390/biology10020106

**Published:** 2021-02-02

**Authors:** Lorenzo Polimeno, Maria Teresa Lisanti, Margherita Rossini, Edoardo Giacovazzo, Lucrezia Polimeno, Lucantonio Debellis, Andrea Ballini, Skender Topi, Luigi Santacroce

**Affiliations:** 1 Polypheno Academic Spin Off, University of Bari “A. Moro”, 70124 Bari, Italy; lorenzo.polimeno@uniba.it (L.P.); lucrezia.polimeno@outlook.com (L.P.); luigi.santacroce@uniba.it (L.S.); 2 Department of Biosciences, Biotechnologies and Biopharmaceutics, Campus Universitario “Ernesto Quagliariello”, University of Bari Aldo Moro, 70125 Bari, Italy; mtlisanti23@gmail.com (M.T.L.); edoardogiacovazzo@gmail.com (E.G.); lucantonio.debellis@uniba.it (L.D.); 3 Clinical Pathology Unit, Policlinico University Hospital of Bari, 70124 Bari, Italy; margherita.rossini@policlinico.ba.it; 4 Department of Precision Medicine, University of Campania “Luigi Vanvitelli”, 80138 Naples, Italy; 5 Department of Clinical Disciplines, University of Elbasan “A. Xhuvani”, 3001 Elbasan, Albania; skender.topi@uniel.edu.al; 6 Department of Interdisciplinary Medicine, Microbiology and Virology Unit, Policlinico University Hospital of Bari, University of Bari Aldo Moro, 70124 Bari, Italy

**Keywords:** gastro-allergic anisakiasis, aquacultured fish, food allergy, clinical microbiology, public health, clinical molecular biology, fishmeal, *Anisakis* allergy

## Abstract

**Simple Summary:**

The diagnosis of Anisakiasis is documented by the occasional finding of L3 larvae in the infected gastro-intestinal tract. Currently, about 14 allergens have been described, among which Ani s1 and Ani s4, both highly heat-resistant, appear central in Anisakiasis anaphylaxis and necessary to cause allergic reactions. Food has to be considered *Anisakis*-free only when heat-resistant *Anisakis* allergens are not present.

**Abstract:**

Background: *Anisakis simplex* (*A. simplex)* infection, in humans, causes a series of clinical manifestations affecting the gastro-intestinal tract known as Anisakiasis/Anisakidosis. Patients may also present allergic manifestations such as hives and/or angioedema and even anaphylactic shock. The aim of this study was to investigate whether aquacultured fish could be considered *A.* *simplex*-free food and constitute a safe, alternative, wild-capture fish food for Gastro-Allergic Anisakiasis (GAA)-sensitized subjects. Methods: Protein extracts from *A. simplex* larvae in the third stage (L3) and from edible part of heavily infected horse mackerel (*Trachurus trachurus*) and aquacultured sea bream, have been tested for *A. simplex* allergens presence by immunological analysis. Western blot analysis using, as source of specific *Anisakis* allergens antibodies, serum samples from subjects referring allergic symptoms after raw fish ingestion, was performed. These subjects showed high levels of specific IgE anti *A.* *simplex* allergens determined by clinical laboratory tests (ISAC test). Results: Our data demonstrate the presence of Ani s4 allergen in both infected and aquacultured fish extracts, providing a possible interpretation for the allergic manifestations reported by subjects, already sensitized to *A. simplex*, who ate frozen or well-cooked or, even, aquacultured fish. Conclusions: The present data stimulate more accurate prophylaxis suggestions for *Anisakis* allergy and more specific controls of fishmeal used in aquaculture.

## 1. Introduction

There are numerous zoonotic diseases in humans that can be transmitted by ingestion of parasite-infected foods. Among the parasites responsible for indirectly transmitted zoonoses are nematodes which, found in a wide range of marine organisms, play a decisive role, in this context, especially for those populations accustomed to consuming marine products [1,2,3,4,5,6]. At the same time, the changes in food tastes that have characterized humans’ eating habits in recent years have increasingly directed consumption towards fresh and natural products, and the introduction of habits and culinary specialties from different countries has also led to an increase in the consumption of raw fish products [4].

*Anisakis simplex* (*A*. *simplex*) is a nematode which, at its third-stage larvae (L3), can infect humans [7] eating raw, undercooked and even smoked, salted or in brine parasitized fish (cod, tuna, sardines, anchovies, salmon etc.) or cephalopodos [8]. Parasite specimens have been detected also in a number of uncommon hosts including Grey petrel, *Procellaria cinerea*; Little penguin, *Eudyptula minor*; Blue-lipped sea krait, *Laticauda laticaudata* and Spinner shark, *Carcharhinus brevipinna* [9] as well *Delphinus delphis*, *Tursiops truntatus* and *Kogia sima* [10].

*A. simplex* infection causes a series of clinical manifestations affecting the gastro-intestinal tract known as Anisakiasis/Anisakidosis; *A. simplex*-infected patients may have, in addition to abdominal symptoms, allergic manifestations such as urticaria and/or angioedema and even anaphylactic shock [1]. Reports are increasingly numerous claiming the appearance of allergic symptoms, after ingestion of fish, such as canned fish, in subjects previously *A. simplex*-infected [5,6]. This indicates that the prophylaxis currently suggested, fish cooked at 60 °C for 10′ or frozen at −20 °C, for at least 24 hr, might not be sufficient to avoid the allergic effects caused by infested fish ingestion, probably due to the thermal-resistance of *A. simplex* allergens [5]. Indeed, it is known that several *A. simplex* allergens are heat stable, supporting the hypothesis that cooking or freezing procedures may not protect humans against allergic reactions as observed when the parasite is subjected to high temperature conditions or to the thermal procedures adopted for marine food preparation [11].

Furthermore, much debated is the question of whether, for the induction of allergic manifestations, the viable L3 larvae ingestion is necessary [12] or if just the exposure to parasite allergens can trigger adverse reactions even when L3 larvae are killed by freezing and/or cooking the fish [13]. Some *A. simplex* allergens show to be relatively resistant to enzyme digestion or heat treatment: Ani s1 [14]; cystatin Ani s4 [15] and allergens belonging to the SXP/Ral family such as Ani s5 [16], Ani s8 [17] and Ani s9 [18]. These proteins could account for the occurrence of hypersensitivity reactions to fishery products contaminated by *A. simplex* proteins. The accidental presence of these allergens in food would recommend to *A. simplex*-sensitized patients to avoid the use of fishery products resulting from (i) losing the beneficial nutritional effects (*ω*-3 lipids and proteins with high biological effect) [19,20] and (ii) causing negative commercial relevance to high income populations through marine products. Two possibilities seem to circumvent such a problem: (i) removal of *A. simplex* allergens from infected fish or (ii) eating *A*. *simplex* allergens-free fish. Olivares et al. reported that in order to remove *A. simplex* allergens from infected fish, for example in the preparation of surimi following the presence of the heat resistant allergen Ani s4, several washing steps with water and strong buffers are required, making this process impractical [21]. Therefore, the removal of *A. simplex* allergens from marine products not being convenient or safe, for previously sensitized patients by L3 *A. simplex* allergens, eating *A. simplex* allergens-free aquacultured fish seems to be the only suitable way to avoid the allergic manifestations caused by them [4,22]. On the other hand, as stated by Fæste et al., the detection of immunoreactive *Anisakis* peptides in the tissue of zebrafish exposed to high amounts of *A. simplex* in the feed can be regarded as a proof-of-principle that allergenic peptides may be transferred from animal feed into the final food products [22].

The growth of the fish aquaculture industry has outpaced production of wild-capture fisheries for over 2 decades, currently producing nearly 50% of all seafood consumed globally. As wild-capture fisheries continue to decline, aquaculture’s role in food production will grow, and it will produce an estimated 62% of all seafood consumed in 2020 [20,21]. The feeding of fish in aquaculture usually consists of flours obtained by adding fishmeal in order to increase the efficiency and growth of the animals [23]. The balanced amino acid composition of fishmeal integrates and quickly promotes growth and reduces feeding costs. In addition, fishmeal provides a balanced amount of all essential amino acids, phospholipids and fatty acids (docosahecsenic acid-DHA and eicosapentaenoic acid-EPA) [23].

Previous studies reported a minor presence of Anisakide parasites in farmed fish, suggesting that consumption of these fish species carries almost no risk of exposure to these nematodes [24,25,26,27]. The aim of the present study was to evaluate the presence of heat-resistant allergens in aquacultured fish, fed with a standard fishmeal-based diet. The data obtained were compared with similar data from wild-capture L3 *A. simplex* larvae-infected fish and, as positive control, protein extracts from L3 *A. simplex* larvae. An immunological procedure, Western blot analysis, was set up, using as *A. simplex* allergens antibodies, serum samples from subjects previously accidentally sensitized by *A. simplex* allergens against which high sIgE levels were evidenced by a commercial microarray immunoassay.

## 2. Materials and Methods

The study was conducted in agreement with the ethical guidelines of the Declaration of Helsinki and received the approval of the Ethical Committee of the University Elbasan (INTL_ALITMKCOOP/HealthMicroPath/HMM2019_IPM).

Serum samples for Abs anti-*A. simplex* allergens were obtained from patients with allergic and/or gastrointestinal symptoms within 12 hrs after ingestion of fish. Seventeen subjects were enrolled during the study period (March–November 2019). Specific immunoglobulin (sIgE) to *A. simplex* allergens was evaluated by an allergen microarray immunoassay (*ImmunoCAP**^®^** Specific IgE* Phadia, (ThermoFisher Scientific, Milan, Italy), a fluoro-immunoassay, repeatable and reproducible in vitro diagnostic tool for sIgE determination that allows detection of sIgE to 112 molecular components from 51 allergenic sources. All selected serum samples for the study showed undetectable sIgE to fish and in particular to cod, shrimp and dust mite. The negative limit for *A. simplex* sIgE, for *ImmunoCAP**^®^*** is ≤0.3 Standardized Units (ISU-E), and all the serum samples selected showed sIgE levels mainly between 1 and 15 ISU-E. The serum samples induced a strong positivity when used, preliminarily, as Abs source in a dot-blot analysis performed with L3 *A. simplex* larvae as antigens (data not reported). All subjects included in the study were asked to sign an informed consent form for the use of their serum samples which, alternatively, would have been safely eliminated.

### 2.1. L3 Anisakis Simplex Larvae

(a) Extraction of L3 larvae. *A. simplex* L3 larvae were obtained from heavily infected *Trachurus trachurus*, called horse mackerel, from a fish market in Bari (Italy). Even if it has been reported that *Trachurus trachurus* is infected with Hysterothylacium larvae [28,29], in our study, *A. simplex* L3 larvae were identified. For each protein preparation, 400 to 500 L3 larvae were used. The L3 larvae were washed in PBS, collected and stored, in PBS, frozen at −20 °C until use. The L3 larvae were, morphologically, identified as *Anisakis simplex sensu lato* by one of the investigators (L.D.).
(b) Protein preparation. *A. simplex* L3 larvae were subsequently ground in a Potter-ELV homogenizer in a RIPA-buffer (TRIS HCl 25 mM, NaCl 150 mM, 1% Triton x-100, Sodium Deoxycholate 1%, SDS 0.1%) with anti-proteases and sonicated at 103 18 w for 5 s. The homogenate was then centrifuged at 16,000× *g* for 10 min. The protein extract was then aliquoted in 200 µL Eppendorf tubes and frozen at −20 °C until the use, mainly on the day after protein preparation. The protein concentration was determined by using Quick Start 105 Bradford Protein Assay (Bio-Rad Laboratories S.r.l., Milan, Italy) and using bovine serum albumin (BSA) as protein standard.

### 2.2. Aquacultured and Infected Fish

(c) Protein preparation. Five aquacultured sea bream and five L3 *A. simplex* larvae-infected horse mackerel, previously stored at −20 °C for 24 h, were utilized for the study. Protein preparation was conducted mainly following the procedure used for L3 *A. simplex* larvae. Of each fish, 2 g of edible part was weighed and homogenized with 7 mL of RIPA buffer (Sigma-Aldrich, Milan, Italy), containing protease inhibitors cocktail tablets (Roche Applied Science, Milan, Italy) and anti-phosphatases (sodium orthovanadate 2 mM; Sigma-Aldrich, Milan, Italy). The homogenate was then centrifuged at 16,000× *g* for 30 min and separately aliquoted and stored at −20 °C until the use.

### 2.3. Western Blot Analysis

The procedure was done mainly as suggested by Rodriguez-Mahillo et al. [15]. Briefly, proteins samples, respectively from 3 protein extracts of *A*. *simplex* larvae, 5 protein extracts of sea bream and 5 protein extracts of *Trachurus trachurus* were used. Aliquots of 20 µg were separated on 4–12% sodium dodecyl sulphate polyacrylamide gels (Invitrogen S.r.l., Milan, Italy), transferred onto a nitrocellulose membrane, later incubated for 1 h in a blocking solution (5% of non-fat dry milk) (Bio-Rad Laboratories S.r.l., Milan, Italy) in TBS-T and then incubated, overnight at 4 °C, under shaking, with a pool of *A. simplex* allergic patients’ sera diluted 1:10 in blocking solution. The primary antibody was identified by an HRP-conjugated secondary antibody, anti-human immunoglobulin (Bio-Rad Laboratories S.r.l., Milan, Italy), diluted 1:20,000 in TBS-T and subsequently detected by a chemiluminescent substrate of HRP (Pierce Biotechnology, Inc., Rockford, IL, USA). The chemiluminescence analysis of each signal on nitrocellulose membranes was evaluated by Molecular Image Chemidoc XRS+ (Bio-Rad Laboratories S.r.l.) as by us previously reported [30]. Each analysis was done, at least, three times, using, each time, a different protein extract. 

### 2.4. Dot-Blot Analysis

The aim of this procedure was initially adopted to identify serum samples from *A. simplex* sIgE positive subjects, evaluated by *ImmunoCAP^®^ Specific IgE* analysis, able to ensure their use for Western blot analysis. Afterwards, this procedure was used to test the immunological presence of *Anisakis* allergens in commercial flours normally utilized in aquaculture. Protein extracts, carried out three times from two different flours, were loaded on nitrocellulose membranes contemporary to protein extracts of *A*. *simplex* L3 larvae. The nitrocellulose membranes were then treated with blocking solution (TBS-T, TRIS-HCl 20 mM, NaCl 150 mM, Tween 20 0.05%, pH 7.5) for 30 min, incubated with the human serum cocktail in TBS-T (Tris Buffer Saline Tween 20) overnight at 4 °C, washed with TBS-T and finally incubated with an anti-IgE antibody solution in TBS-T for 1 hr at room temperature. To verify the antigen-primary antibody-secondary antibody reaction, the membranes were incubated with a chemiluminescent substrate for 5 min, and the chemiluminescence on nitrocellulose membrane evaluated by ChemiDoc XRS+ (Bio-Rad Laboratories S.r.l., Milan, Italy) as above described and by us reported (30). Each analysis was done at least three times, using, each time, a different protein extract.

## 3. Results

**Figure 1:**
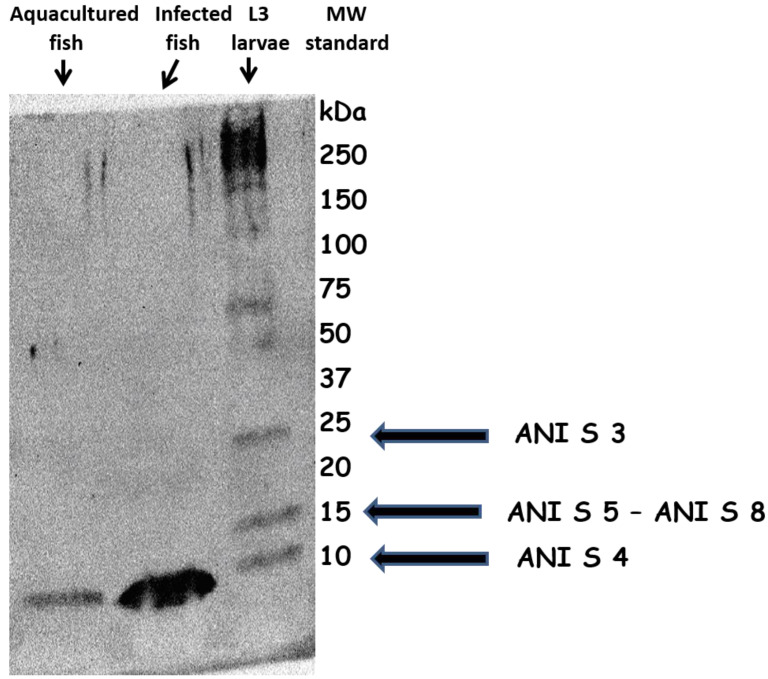
WB analysis of protein extracts from aquacultured fish, *A. simplex* L3 larvae-infected fish and *A. simplex* L3 larvae. MW identification shows, in the edible part of both fishes, the presence of an immunodetected protein signal similar to the Ani s4 allergen of *A. simplex* L3 larvae.

Figure 1 reports Western blot analysis of protein extracts from aquacultured (sea bream), L3 larvae-infected fish (horse mackerel) and *A. simplex* L3 larvae. In both fish extracts it is possible to observe an immunological protein signal below 10 Kd MW, that parallels the L3 larvae signal with similar MW. As it is well known, this MW characterizes Ani s4 *Anisakis* allergen, a heat and pepsin-resistant allergen. The Figure is an example of WB analyses carried out running all protein extracts. Every time, an immunological signal below 10 Kd MW, not always with the same intensity, was evidenced.

**Figure 2:**
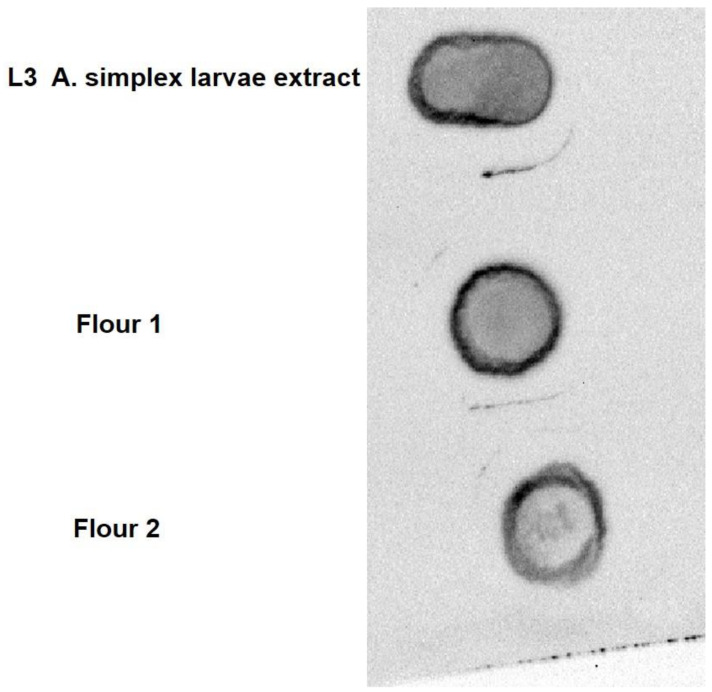
Dot blot analysis of protein extracts from *A. simplex* L3 larvae and two commercial flours widely used for feeding fish and chicken destined for human nutrition.

Figure 2 reports dot blot analysis of two commercial flours widely used as animal feed for human food, such as farmed fish or poultry. In the Figure the immunological analysis using protein extract from *A. simplex* L3 larvae is also reported. A positive signal was detected in all three protein samples, evidencing the possibility of the presence of antigenic proteins recognized by the sera of *A. simplex* allergens positive patients. Constantly, a positive immunological signal was highlighted, testing, each time, a different protein extract of the two flours. 

## 4. Discussion

The results herein reported show an evident allergenicity for *A. simplex* allergens in the edible part of both *A. simplex* L3 larvae-infected and in aquacultured fish, farmed in shore cages and fed with commercial flour. It is important to underline that previous studies reported almost no risk of exposure to anisakid parasite considering the very low presence of these nematodes in farmed fish [24,25,26,27]. Similarly, no anisakid nematode was detected in the aquacultured fish by us analyzed. However, the tested flours, immunologically positive to human sera from patients with high IgE titers directed to *Anisakis* allergens (Figure 2), were the same used to feed the aquacultured fish.

Considering the molecular weight of the protein signal evidenced in all protein extracts (Figure 1), the detected *A. simplex* allergenicity seems to be determined by the presence of Ani s4, a heat and pepsin-resistant allergen. 

Ani s4, the *A. simplex* allergen detected in 27% of allergic patients, is the first nematode protease inhibitor (cystatin) described as an allergen and has been previously shown to be heat stable (boiling for 30 min) and resistant to pepsin digestion [15]. Its resistance to autoclaving, along with pepsin resistance, suggests that Ani s4 could be clinically relevant in cases in which Ani s4–sensitized patients are again exposed to *A. simplex* allergens following consumption of processed parasite-contaminated fishery products [11,31].

Our data may provide an interpretation for the allergic manifestations to *A. simplex* reported by subjects who consumed aquacultured or previously frozen fish and, in the same patients, symptom presentation was reported coincident with an increase of specific IgE level against *Anisakis* allergens (4). Similarly, Armentia et al. reported allergic reactions in patients highly sensitized to *A. simplex*, eating chicken meat fed with fishmeal [32]. We hypothesize that, although seafood is the principal source of human infections by *A. simplex*, it may be possible that flours used to feed animals for human food, such as farmed fish or poultry, are contaminated with nematode allergens resistant to the treatments for their preparation. Despite the exceptions reported to the Armentia study [33,34], the problem of the possible presence of heat-resistant anisakide allergens in animal food remains. We believe that with our data, we partially clarified this topic.

The clinical and epidemiological interest in *A*. *simplex* allergens’ thermostability therefore plays a crucial role in suggesting the prophylaxis against *A. simplex* (fish cooked at 60 °C for 10′ or frozen at −20 °C for at least 24 h), neglecting any reference not only to the thermostable proteins of *A. simplex* but also to the healthiness of fishmeal widely used in aquaculture or in chicken feed. 

The feeding of fish in aquaculture usually consists of flours added with fishmeal in order to increase the efficiency and growth of the animals. Fishmeal provides a balanced amount of all essential amino acids, phospholipids and fatty acids (docosahecsenic acid-DHA and eicosapentaenoic acid-EPA) [23]. Usually, aquaculture industry feeds fish using fishmeal, even if a number of carnivorous and omnivorous farmed fish species are capable of digesting poultry meals, nuts, soy, and grain on commercial scales [35,36,37], presenting the possibility that fishmeal can be eliminated as a component of fish feed. Previous studies have tested the feasibility of fishmeal-free feeds by examining how they impact different performance metrics, including growth [38,39,40], palatability [41,42], nutrition [36], fatty acid composition of the fillet [42,43] and water quality [39,44].

Fishmeal preparation usually follows steps which include boiling the raw material and the solid part being dried at about 80–100 °C, a temperature which must not be too high, and the process must last as short as possible in order not to destroy the proteins [34]. Moreover, as reported by Miles and Chapman, the top fishmeal producing countries are Peru, Chile, China and Thailand, and most of the fishes used to produce it are small, bony, with high content of oil and considered of little edible use (e.g., anchovies, herrings, capelin and menhaden). A small percentage of fishmeal is rendered from fish offal, trimmings or cuttings and other wastes principally from filleting and canning operations from the edible fisheries (e.g., tuna, cod, haddock, hake, pollock) [45,46].

As previously reported, the aquaculture industry has outpaced wild-capture fisheries for over two decades and currently produce nearly 50% of all seafood consumed globally. As wild-capture fisheries continue to decline, aquaculture’s role in food production will continuously grow [47,48,49].

All these lead us to consider more carefully the use of foods that are used by the farms dedicated to producing food for human consumption, and not only in the field of aquaculture, taking into consideration the possibility of allergic phenomena triggered by *A. simplex*. In this regard, as we previously reported, an ever-increasing number of observations report allergic phenomena in subjects who, perhaps, do not eat raw or undercooked seafood products or for whom, furthermore, fish is not part of their eating habits [4]. Our research also highlights the need for a one-health approach to increasing the awareness among stakeholders, including fish farmers, food manufacturers and fisheries authorities for change in policies and protocols for a safer seafood production [50].

## 5. Conclusions

In conclusion, we believe that in order to avoid the threats to human health deriving from allergic episodes caused by the ingestion of infected marine products, to preserve the possibility of being able to consume a nourishment such as fish, which rich in *ω*-3 lipids and proteins of high biological value, it is no longer possible to suggest the useless prophylaxis against *A. simplex*, which involves freezing and/or boiling the fish, but checks must be carried out on the foods used for aquacultured fish, as well as for the production of poultry, intended for human consumption, an industry which, so far, covers nearly 60% of all seafood consumed globally.

## Data Availability

The data presented in this study are available on request from the corresponding author.
